# Software as a Medical Device (SaMD) in Digestive Healthcare: Regulatory Challenges and Ethical Implications

**DOI:** 10.3390/diagnostics14182100

**Published:** 2024-09-23

**Authors:** Miguel Mascarenhas, Miguel Martins, Tiago Ribeiro, João Afonso, Pedro Cardoso, Francisco Mendes, Hélder Cardoso, Rute Almeida, João Ferreira, João Fonseca, Guilherme Macedo

**Affiliations:** 1 Precision Medicine Unit, Department of Gastroenterology, São João University Hospital, 4200 427 Porto, Portugal; miguel.pedro96@gmail.com (M.M.); tiagofcribeiro@outlook.com (T.R.); joaoafonso28@gmail.com (J.A.); pedromarilio@gmail.com (P.C.); francisco.cnm@gmail.com (F.M.); hc@sapo.pt (H.C.); guilhermemacedo59@gmail.com (G.M.); 2 WGO Gastroenterology and Hepatology Training Center, 4200 427 Porto, Portugal; 3 Faculty of Medicine, University of Porto, 4200 427 Porto, Portugal; 4 CINTESIS@RISE, Department of Community Medicine, Information and Health Decision Sciences (MEDCIDS), Faculty of Medicine of University of Porto, 4200 427 Porto, Portugal; rutealmeida@med.up.pt (R.A.); fonseca.ja@gmail.com (J.F.); 5 Department of Mechanic Engineering, Faculty of Engineering of University of Porto, 4200 427 Porto, Portugal; jferreira@fe.up.pt; 6 DigestAID—Digestive Artificial Intelligence Development, 4200 427 Porto, Portugal

**Keywords:** software, medical device, gastroenterology, digestive healthcare, artificial intelligence

## Abstract

The growing integration of software in healthcare, particularly the rise of standalone software as a medical device (SaMD), is transforming digestive medicine, a field heavily reliant on medical imaging for both diagnosis and therapeutic interventions. This narrative review aims to explore the impact of SaMD on digestive healthcare, focusing on the evolution of these tools and their regulatory and ethical challenges. Our analysis highlights the exponential growth of SaMD in digestive healthcare, driven by the need for precise diagnostic tools and personalized treatment strategies. This rapid advancement, however, necessitates the parallel development of a robust regulatory framework to ensure SaMDs are transparent and deliver universal clinical benefits without the introduction of bias or harm. In addition, the discussion highlights the importance of adherence to the FAIR principles for data management—findability, accessibility, interoperability, and reusability. However, enhanced accessibility and interoperability require rigorous protocols to ensure compliance with data protection guidelines and adequate data security, both of which are crucial for effective integration of SaMDs into clinical workflows. In conclusion, while SaMDs hold significant promise for improving patients’ outcomes in digestive medicine, their successful integration into clinical workflow depends on rigorous data protection protocols and clinical validation. Future directions include the need for adequate clinical and real-world studies to demonstrate that these devices are safe and well-suited to healthcare settings.

## 1. Introduction

A major shift that defines this century has been the increasing reliance on software in all spheres of life, and healthcare has not been exempt from this movement. The digitalisation of healthcare has brought major changes, particularly in medical imaging, resulting in significant benefits. The software used in healthcare may be integral to, or used in, the manufacturing/maintenance of a medical device, or as stand-alone software as a Medical Device (SaMD). In this manuscript, we will focus on SaMDs, specifically those intended for medical purposes such as the diagnosis, prevention, monitoring, or treatment of disease [1]. (https://www.fda.gov/medical-devices/digital-health-center-excellence/digital-health-terms; https://www.ema.europa.eu/en/human-regulatory-overview/medical-devices, accessed on 17 July 2024), with an emphasis on their application in digestive medicine.

There are several examples where SaMDs are making a significant impact in digestive healthcare, contributing to improved diagnosis, e.g., artificial intelligence (AI) assisted endoscopy, virtual colonoscopy software, AI-powered analysis of stool samples, or enhanced patient treatment and management (monitoring digestive health, dietary management, telemedicine platforms, for remote consultations) [2,3,4]. These applications offer the potential for early detection, more personalized care, enhanced patient engagement, and streamlined workflows [5], providing both patients and healthcare professionals with powerful tools to achieve earlier diagnoses, better treatment options, and, ultimately, improve patient outcomes [6,7].

The emerging field of AI and big data, marked by a rapid technological advancement, presents a unique challenge: balancing innovation with ethical considerations, while ensuring everything operates for the benefit of humanity. Due to the field’s early stage, its full potential remains speculative, which has made the development of effective regulatory frameworks inherently complex. The ethical and regulatory problematic should be a key concern for gastrenterologists, as addressing it will help build ethical oversight while simultaneously facilitating the continued progress of technological development.

This manuscript provides the first in-depth analysis of the regulatory and ethical challenges of SaMDs in digestive healthcare, highlighting both the current state of the art and the complexities of their implementation. The structure of the manuscript is as follows: we will first describe the evolution of these devices, assess the barriers they must overcome, revisit key technologies that form the foundation of these devices, discuss their integration into clinical practice, and examine their potential clinical impact. We will then explore the regulatory and ethical considerations they must adhere to. Finally, we will explore future innovations and advancements that may shape the next generation of SaMD in digestive healthcare.

## 2. Evolution of SaMDs in Healthcare

### 2.1. Milestones of SaMDs in Healthcare

The first inclusion of software into the clinical environment likely dates back to the early 1950s, related to administrative tasks (hospital billing/accounting) and data management (recording/storage of patient information). Over time, the latter has evolved into more sophisticated applications, such as today’s Electronic Health Records (EHRs) [8,9]. Developments in informatics have enabled medical software to evolve dramatically. Since the introduction of the first EHRs (software designed for laboratory analysis and other clinical tasks) in the 1980s, medical software has advanced tremendously, now encompassing diagnostic tools and decision support systems, software for image analysis (X-rays, MRIs, etc.), telemedicine and remote patient monitoring, and even the education and training of medical professionals.

As a result, software has become an essential part of modern medicine, driving more accurate diagnosis and more effective patient management. Digital imaging, in particular, has revolutionized the field of medical diagnostics, offering significant advancements in resolution and image quality. These improvements not only enhance diagnostic efficiency but also contribute to better patient outcomes. Additionally, the enhanced capacity to store and share digital images has proven invaluable for training and education. These developments are particularly relevant to digestive healthcare, a speciality that relies heavily on imaging techniques for diagnosis and monitoring. Digitalization has introduced advanced informatics solutions for managing and analysing medical images, further benefiting this field. Another advantage of digital images is the ability to use semi-automated and even fully automated analytical software tools, which can be integrated into SaMDs.

### 2.2. Overview of Significant Breakthroughs Specific to Digestive Health

The most important breakthrough in digestive healthcare occurred in the early 20th century with the development of gastroscopy and colonoscopy. These two procedures have since become the cornerstone of this specialty, as they enable the direct visualization of the gastrointestinal (GI) tract, allowing for the detection of lesions and changes in these tissues [10]. These techniques were further improved by the incorporation of fibre optics, which enhance image quality, and the advent of laparoscopic surgery towards the end of the century, which introduced minimally invasive procedures. These advancements significantly expanded the impact of these innovations in managing digestive problems.

The benefits of minimally invasive interventions have been further enhanced by the introduction of capsule endoscopy (CE), arguably the most important breakthrough since the initial development of gastroscopy techniques [11]. CE is a non-invasive and painless technique that allows the visualization of the entire small intestine, including regions inaccessible to standard techniques. As an outpatient procedure, CE led to improved detection rates, particularly for conditions like Crohn’s disease (CD) or obscure GI bleeding (bleeding from an unknown source) [12,13,14].

CE also produces digital images with superior resolution, which can be analysed post-procedure, offering flexible assessment options, and they can be easily stored and shared. Given the enormous amount of data generated by this technique, its potential can be maximized by integrating automated image processing and analytical methods into the CE routine.

The rise in the use of software in digestive healthcare has been further bolstered by the incorporation of the latest developments in the field, including that of Artificial Intelligence (AI) [15,16].

### 2.3. The Rise of Software as a Medical Device (SaMD) in Digestive Healthcare

The incorporation of digital images and the increased use of CE in digestive healthcare has been accompanied by a rising application of SaMD. SaMD may be incorporated into the endoscopy apparatus, enabling the control of the capsule or image capture, assisting in lesion detection, or assessing the quality of bowel preparation—a fundamental factor for ensuring diagnostic efficiency. The integration of advanced algorithms like SaMD has led to exciting developments, including significant enhancements in endoscopic procedures. Among these advancements, the incorporation of AI algorithms—especially machine learning (ML)—is particularly noteworthy. These AI-driven methods enable real-time image analysis, thereby improving both accuracy and efficiency [17]. Furthermore, by reducing dependence on the endoscopist’s experience and visual acuity, which can vary considerably, these algorithms help standardize and enhance diagnostic precision.

Another exciting development associated with the introduction of specific software is virtual colonoscopy, where software generates a detailed 3D image of the colon from X-ray images obtained through a CT scan [18]. This procedure is relatively fast and does not require sedation. As a non-invasive technique, it is effective at detecting abnormalities such as polyps or lesions, although it may not be well suited for certain types of lesions. Moreover, unlike traditional colonoscopy, it is not possible to obtain a biopsy for further investigation, and the exposure to radiation (albeit low-dose) may not be appropriate in all cases.

## 3. Data Science Technological Framework

### Description of the Key Technologies Underpinning SaMD—AI, Machine Learning, Big Data Analytics, Cloud Computing

**Figure 1:**
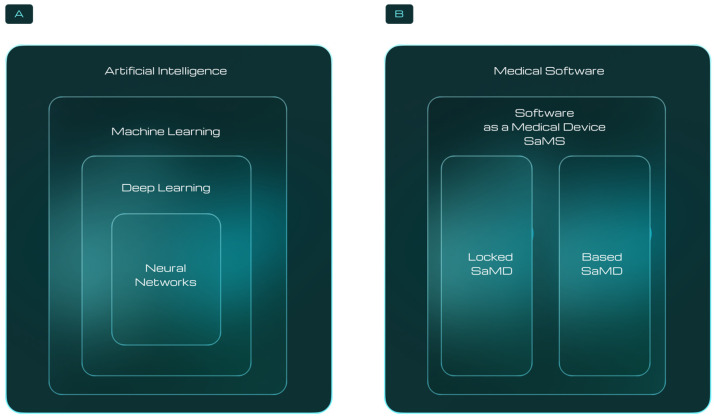
(**A**) The types and hierarchy of AI algorithms suitable for use in digestive healthcare applications. (**B**) Software tools applicable in digestive healthcare can be categorized as either Locked SaMDs (static devices which produce the same result when given the same input—e.g., PillCam’s Top 100) or AI-based SAMDs (which can learn and adapt, potentially improving their performance over time—e.g., Medtronic GI Genius).

Since the initial appearance of SaMDs in digestive healthcare, these technologies have evolved considerably, with perhaps the most important changes in recent years being the introduction of AI and the advances in computational capacity, particularly in cloud computing and Big Data analytics (e.g., the vast accumulation of digital images). The term AI covers a range of disciplines, and in the context of medical imaging it is more specifically on the ML class of AI tools most commonly used to recognise patterns in complex datasets (see Figure 1 [17]). ML initially involves setting up an algorithm (including data preparation, feature engineering, and the selection of relevant features) to evaluate important elements in images. The algorithm then identifies the combination of features that best classify the image or defines a metric for a specific image region.

Deep Learning (DL) is a sub-class of ML based on artificial neural networks, is extensively applied to image analysis due to its artificial resemblance to neurobiological processes, and is inspired by the activity of the human visual cortex. DL performs well in medical applications, accurately detecting and classifying lesions and disease characteristics, thereby enhancing diagnostic efficiency. Multi-layered convolutional neural networks (CNNs) are the DL algorithms best adapted for analysing clinical images. They use multiple layers to progressively extract more detailed features from images, excelling in the analysis, differentiation, and classification of medical images and videos. ML models can be classified as “supervised” or “unsupervised”, with the former involving attempts to predict a known output (e.g., protruding lesion in the small bowel) that are more relevant to current applications in gastroenterology [15,16]. Nevertheless, unsupervised ML holds significant potential in the field of drug discovery in digestive healthcare, as well as in leveraging Big Data in pivotal areas like inflammatory bowel disease (IBD). Given the potential influence of ML in the future, clinicians who rely on medical images in their work must become familiar with how ML functions.

SaMDs are closely linked to Big Data analytics, primarily due to the large amount of data that can be collected from patients (e.g., through EHRs, wearable devices, sensors, or even environmental factors) or through the device itself (e.g., performance metrics, usage patterns, and real-time readings). Meaningful information can be extracted from such data using Big Data analytic tools, identifying patterns and trends in patient health, predicting potential health risks or complications, and optimizing the performance of SaMDs [19,20]. Indeed, Big Data play a crucial role in training ML algorithms used with SaMDs, aiding the analysis of patient data for disease diagnosis and risk prediction, personalizing treatment recommendations, or adapting SaMD use based on real-time data analysis [21]. In summary, Big Data analytics can significantly enhance the performance of SaMDs, thus improving patient outcomes.

It is essential to distinguish between locked SaMDs and AI-based SaMDs in this context. Locked SaMDs are static and produce consistent results from the same inputs, while AI-based SaMDs are dynamic, capable of learning and adapting over time, and potentially leading to ongoing improvements in functionality and performance.

## 4. Clinical Applications and Impact

### 4.1. Case Studies Illustrating the Clinical Impact of SaMD on Patient Care and Treatment Outcomes

A fertile area for the recent development of SaMDs in digestive healthcare has been the design of AI-based tools to analyse endoscopy images, both in real-time and off-line. This disruptive technology can aid in detecting abnormalities such as polyps or lesions, offering greater accuracy and sensitivity than traditional methods. It can also leverage Big Data in conjunction with information from EHRs, generating an unparalleled set of efficient resources (see Table 1). There is increasing evidence of the potential of AI tools to enhance the performance and diagnostic accuracy of GI diagnostic procedures, the future of which will undoubtedly involve ancillary SaMDs.

Taking CE as an example, while AI shows promise for lesion detection, challenges remain in real-time analysis and balancing sensitivity and specificity, as higher sensitivity may lead to more false positives. Nonetheless, the future looks promising for AI integration in CE. Indeed, AI applications addressing a variety of issues in this area have been evaluated, such as the detection and classification of various types of lesions (e.g., ulcers/erosions, vascular lesions, protruding lesions, and blood), not only in the small bowel but also in the colon [22,23,24]. Furthermore, DL approaches have been employed to develop scoring systems that help stratify risk, and predict prognosis or response to treatment, for example by predicting patient survival after hepatocellular carcinoma resection [25] or identifying which IBD patients might benefit from biological therapies [26]. Similarly, AI can be used to evaluate parameters such as the quality of bowel preparation or of the endoscopic images obtained. As a proof of concept, a pioneering AI solution was developed to detect, differentiate, and stratify the haemorrhagic risk of SB lesions in CE images, marking an important step towards the incorporation of AI in CE. It should be noted at this point that the possible use of AI algorithms for the automated analysis of endoscopy images recently led to the approval of the first system for clinical use by regulatory agencies [3,27].

Recent interest has also emerged for whether SaMDs can be used to analyse images of stool samples, flagging potential issues like blood or parasites for more rapid diagnosis and perhaps enhancing diagnostic accuracy. Indeed, a system was recently proposed to detect occult blood and analyse the gut microbiome through stool samples collected from toilets [28]. Software can be trained with large datasets of stool images to identify these abnormalities, which can then analyse user-uploaded images and flag potential issues. Significantly, such tools might facilitate remote stool analysis, improving access to healthcare in underserved areas. The recent FDA approval of a SaMD in the GI space, Geneoscopy’s ColoSense test of RNA markers for colorectal cancer screening, paves the way for future AI-based tools for stool analysis [29].

Virtual colonoscopy already uses software to analyse the 3D model of the colon generated from CT scans, which can then be examined for the presence of polyps and other abnormalities, e.g., Siemens Healthineers syngo.CT Colonography [29] and the Vitrea Workstation by Canon Medical Systems [30]. This approach is fast, avoiding the need for sedation, with a lower risk of bleeding or perforation. It may also be a better option for patients with some health conditions that make traditional colonoscopy risky. However, virtual colonoscopy may miss some polyps, especially flat ones, and a conventional colonoscopy will still be necessary to confirm potential alterations and to obtain tissue biopsies if required.

### 4.2. The Integration of SaMDs into Clinical Workflows and Healthcare Systems

In terms of GI diseases, there is clear evidence that incorporating SaMDs into the clinical workflow can improve patient diagnosis, treatment, and management. There are several ways this can occur, and these methods are constantly being explored and improved, with some SaMDs being currently in use in the clinical practice as part of imaging systems, as explained above. A notable area reflecting this is the development of mobile apps to monitor digestive health [31]. These apps allow patients to track symptoms, bowel movements, and medication adherence, empowering patients to manage their health. SaMDs can also help create personalized meal plans, promoting gut health and managing digestive issues like IBD.

SaMD-based telemedicine platforms facilitate remote consultations with gastroenterologists, improving patient access to care, especially for those who are far from specialized settings or have limited mobility. As a result, these tools can help reach more accurate and rapid diagnoses or earlier interventions, potentially leading to more personalised treatment options and enhance their outcomes. These apps generally incorporate educational software, empowering individuals to take a more active role in managing their digestive healthcare, and they can be readily coupled to AI-powered chatbots for symptom evaluation and triage.

In addition, the use of SaMDs to manage administrative tasks and data handling at healthcare facilities should not be ignored, as this can also improve clinical outcomes and reduce costs. Overall, SaMDs represent powerful tools for healthcare professionals and patients that are revolutionizing digestive healthcare, leading to earlier diagnoses, better treatments, and, hence, improved patient outcomes. In the near future, we are likely to see many advances in this field, including tools designed to perform microbiome analysis to optimize personalized gut health [32].

### 4.3. Technical Considerations in Designing SaMDs for Digestive Health—User Interface, Interoperability, Data Management

Several aspects must be considered when designing SaMDs, the first of which is the need for privacy and safety by design, while usability is essential for acceptability and adequate usage. Requirements should be defined with those in mind.

The interfaces of SaMDs must be straightforward and easy to navigate, even for those with limited experience. This can be achieved by using intuitive icons in a logical layout, clearly understood terms, and by avoiding the presentation of excessive information on the screen. The clarity of the interface can be enhanced by allowing the user to customize what they see according to their needs. User research—the process of understanding the needs, behaviours, and attitudes of users to inform the design and development of products or services—can have a crucial role in defining functionalities and interfaces. Regarding safety, prompts and warnings should be used to avoid common and critical mistakes. In addition to back-end security, access to the interface, data storage, and recall must be controlled. Mechanisms must also be in place through the interface that can overcome or limit the problems associated with the system, software, or networking malfunctions.

Interoperability is a crucial feature of a SaMD because it allows these apps or devices to work seamlessly with different devices and systems. Therefore, SaMDs should use standard communication protocols, data formats (contents), and clinical codification (terminologies) systems that enable them to exchange and use information safely, securely, and reliably across various platforms. This standardization reduces errors and prevents adverse events (AEs), while ensuring that the datasets used are more varied and better reflect the population as a whole. Sharing data to ensure interoperability also increases the need for strong data security measures. Ensuring that SaMDs can work across systems involves many stakeholders, including governing bodies like the EMA (European Medicines Agency) and the FDA (U.S. Food and Drug Administration), hospitals, healthcare providers, manufacturers, standards development organizations, and other interested parties.

In 2017, the FDA issued guidance (Design Considerations and Pre-market Submission Recommendations for Interoperable Medical Devices) on creating safe, effective, and interoperable medical devices [33]. This document outlines design considerations for interoperability and specifies information required for premarket submission and labelling. Reliable interoperable SaMDs necessitate standardized methods that function effectively across all healthcare environments. These harmonized standards may be specific to healthcare, such as the DICOM standard (National Electrical Manufacturers Association (NEMA) PS 3.1—2011 Digital Imaging and Communications in Medicine) for medical imaging and related data [34]. Additionally, they can be more broadly applicable standards that extend beyond imaging and data management, including FHIR and HL7 (Fast Healthcare Interoperability Resources and Health Leven Seven, respectively—comprehensive guidelines for data exchange), OMOP (Observational Medical Outcomes Partnership—standardizes data for observational research), and IHE (Integrating the Healthcare Enterprise—integration and interoperability between different healthcare systems—https://www.ihe.net/, accessed on 26 June 2024). CDIS (Clinical Data Interchange Standards Consortium—https://www.cdisc.org/about, accessed on 26 June 2024) also plays a critical role by establishing standards for clinical research data.

As indicated above, in terms of data management and handling, it is critical that the issue of security and privacy is taken into account when designing SaMDs. These tools may collect and store sensitive patient information, images, and/or data from EHRs, which are not only easy to reproduce but also vulnerable to remote access and to manipulation. This is particularly relevant given the economic incentives for cyberattacks on health-related organisations. Hence, robust cybersecurity measures must be implemented to protect patient data [35]. Measures like de-identification should help maintain privacy, despite the ease with which de-identified data can be re-identified [36]. However, the widespread introduction of SaMDs inherently carries a privacy risk, which might be more acceptable if the benefits of these tools were equally distributed, which is not currently the case. The user interface is a component with increased risk, requiring strong security measures like encryption and access controls. In this sense, the FAIR principles for data management (findability, accessibility, interoperability, and reusability) should be adhered to [37]. Indeed, the ability to track user actions and data changes within the SaMD through adequate log recording is crucial for maintaining a clear record and ensuring accountability. In addition to these measures, communications and centralized databases also represent high-risk areas. To mitigate risks associated with centralized databases, techniques like pseudo-anonymization can be useful.

Another important issue associated with all SaMDs is the need for transparency and traceability. The algorithms that drive these devices must be clear and transparent so that the way they reach their decisions can be readily understood, thereby facilitating troubleshooting when necessary. The traceability of data when using SaMDs and during their development is also a mainstay in terms of their accountability. The need for transparency and traceability places significant demands on data management practices. Personal data used by SaMDs need to be managed safely and securely, and obtaining consent to access and use personal data is a key issue. This is especially challenging given the large volumes of data used to train and validate DL tools. Standard consent models, like opt-in (users actively agree to data use) or opt-out (users must actively decline data use), may not be sufficient given the volume and complexity of the data involved [38]. To introduce AI tools into healthcare effectively, especially in areas like digestive healthcare, novel data protection approaches may be necessary. Traditional data protection methods may not be adequate due to the dynamic and large-scale nature of the data involved. For example, it may be possible to perform certain data processing tasks using non-identifiable or pseudonymized data within centralized databases, or by employing federated models (including federated learning), which reduce the need for direct communication of individual data across diverse healthcare entities.

## 5. Regulatory and Ethical Considerations

### 5.1. Global Regulatory Frameworks for SaMD, with Examples from the FDA, EMA, and Other Bodies

The regulatory framework for SaMDs is still evolving, as SAMDs have yet to be fully mature and the regulatory frameworks vary worldwide. The International Medical Device Regulators Forum (IMDR—https://www.imdrf.org/, accessed on 26 June 2024) is an international group working to harmonize SaMD regulation. They develop guidelines to support innovation and ensure SaMDs are safe and effective globally. IMDRF has established guidelines regarding key definitions for SaMDs, a framework for risk categorization, clinical evaluations, and several other relevant issues.

**Figure 2:**
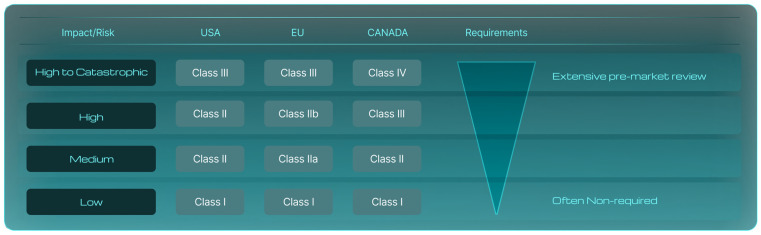
The classification of SaMDs and their regulatory requirements are determined based on the potential risk they pose to patients. This risk can range from benign to life-threatening, influencing the level of scrutiny and regulatory demands necessary for approval. Here is a detailed overview of the different classes and their associated requirements, as well as the underlying logic of these classifications. We present three systems of categorization, namely the American (US), the European (EU), and the Canadian systems.

In the U.S., lower risk SaMDs may go through a more streamlined pre-market notification (510(k)) process, while higher-risk devices require more rigorous premarket approval (PMA) (see Figure 2).

In Europe, regulations from the Medical Device Coordination Group (MDCG—https://health.ec.europa.eu/document/download/dbb9434c-a5ee-497b-be88-44e15ba314e2_pt?filename=md_tor_wg7_nt_en.pdf, accessed on 26 June 2024) also clarify SaMDs by risk and require specific assessments for medium- and high-risk devices. The European Commission has also developed EUDAMED, an IT system designed to implement Regulation (EU) 2017/745 on medical devices and Regulation (EU) 2017/746 on in vitro diagnostic medical devices, which include SaMDs.

For SaMDs intended for treatment, diagnosis, or driving clinical management, a notified body must fully certify the device. Specific SaMD regulation in Europe include MDCG 2019-11, which offers guidance on the qualification and classification of software in Regulation (EU) 2017/745—MDR, and MDCG 2023-4, which provides guidance on medical device software intended to work in combination with hardware or hardware components. There is also a movement in Europe to adopt the principles set out by the IMDRF to regulate SaMDs, harmonizing these regulations globally.

Unlike traditional locked SaMDs, which do not change with use and always produce the same result when given the same input, AI-based SaMDs can learn and adapt, potentially improving over time. The transformative potential of such tools led the FDA to develop a framework for their regulation [39], contemplating an important shift towards a total product lifecycle (TPLC) approach that permits iterative improvement while ensuring patient safety. EUDAMED similarly addresses the entire lifecycle of medical devices, including SaMDs. This framework includes:

1. Setting a clear quality standard and best practices throughout the product’s lifecycle;
2. Planning for how changes to the product will be managed;
3. Ensuring transparency and evaluating real-world performance through post-market studies.

### 5.2. Specific Challenges and Requirements for SaMD in Digestive Healthcare

In digestive healthcare, SaMDs face some unique regulatory challenges and requirements relative to traditional medical devices. The specific regulatory requirements for SaMDs depend on their risk classification, with more stringent requirements applied to higher-risk devices used to diagnose serious conditions than to lower-risk tools for monitoring digestive health (see Figure 2 and Table 2). Many SaMDs in digestive healthcare aim to improve diagnostic accuracy and efficiency, and now incorporate AI based tools. Significantly, most AI-enabled medical devices listed by the FDA and marketed in the USA focus on lesion detection in the GI tract, having met the FDA’s premarket requirements, which include a review of the devices’ overall safety and effectiveness (https://www.fda.gov/medical-devices/software-medical-device-samd/artificial-intelligence-and-machine-learning-aiml-enabled-medical-devices#resources, accessed on 26 June 2024). This novel and rapidly evolving technology requires agile regulatory frameworks that can adapt to these innovations, placing demands on developers to stay informed of the latest regulatory updates and guidance documents.

### 5.3. Ethical Concerns Including Data Privacy, Security, and Patient Consent

The ethical issues faced by SaMDs are pertinent to both AI-enhanced and non-AI SaMDs, whether used in digestive healthcare or other clinical settings. Data security and privacy are among the most critical ethical concerns (as mentioned above), given the sensitive nature of SaMDs’ patient information (images obtained through specific examinations and data obtained from EHRs). Robust data security measures must be implemented to protect patient data from unauthorized access, breaches, or manipulation, and must comply with regulations regarding data protection, such as the EU General Data Protection Regulation (GDPR 2016/79: https://eur-lex.europa.eu/eli/reg/2016/679/oj, accessed on 26 June 2024) or the USA Health Insurance Portability and Accountability Act (HIPAA: https://aspe.hhs.gov/reports/health-insurance-portability-accountability-act-1996, accessed on 26 June 2024).

It is essential to avoid any potential for SaMDs to introduce bias into the clinical decision-making process. Bias can be introduced during the training of these devices or through decisions made during their design [40,41,42,43,44]. By nature, ML-based devices identify patterns in the data used to train and validate them, which shapes their performance. Therefore, the value of these tools depends strongly on the quality of the data used to train. If these datasets are incomplete or unrepresentative, the consequences can be significant [45,46]. For example, using a diagnostic test on patients who do not correspond to the target population for which it was designed can introduce spectrum bias [16]. Disadvantaged groups may be underrepresented in early-stage evidence-based medicine [47]. Another type of bias to avoid is overfitting, which occurs when models are too finely tuned to the training data and do not function correctly with other datasets. Using larger and more diverse datasets for training may help avoid this bias by preventing overtraining and simplifying the models themselves. Thus, initiatives will be necessary to ensure the SaMD development complies with ethical standards, such as those aimed at preventing bias in data and algorithms [48,49].

Transferability to other settings is another critical issue for SaMDs and AI tools [50], ensuring that an algorithm or device developed in one environment performs equally well in another, even if it needs to be retrained on data from the new environment. SaMDs must be carefully designed and validated with consideration for different contexts before they are used on routine clinical care [51], and there must be transparency regarding the data sources used to develop these tools. Clinical validation should demonstrate the accuracy, safety, and effectiveness of SaMDs for digestive health applications by comparing them to traditional diagnostic methods already in clinical use. Moreover, SaMDs must integrate seamlessly with the existing IT environments in hospitals and clinics, which might pose challenges given the current variation in compatibility and data standards.

Emergent blockchain technologies could provide a solution to issues surrounding privacy and data handling in healthcare, offering enhanced traceability and efficient management of clinical information [52]. Blockchain technology enables vast numbers of medical records to be securely stored in a decentralized and ordered manner [53], overcoming the barriers to sharing clinical data associated with local storage. The protection afforded by blockchain technology ensures immutability and can help overcome the fragmentation of patients’ medical records, benefiting both patients and clinicians. Indeed, this could also favour secure communication between healthcare professionals, even across centres, at a radically lower cost. Blockchain offers trustworthiness, enhanced privacy, and data traceability. Federated systems, which keep data local and process it in situ, offer an additional layer of data security and privacy.

## 6. Challenges and Barriers

### 6.1. Development Challenges

Some technical challenges must be considered and addressed when developing useful SaMDs, such as accuracy, reliability, and their dependence on high-quality data. The primary use of SaMDs in digestive healthcare is for image analysis to identify significant visual cues. To ensure these tools are sufficiently accurate, they must undergo extensive validation, particularly using real-world data. This is especially important for ML/DL models to ensure they are valid and can be applied to new datasets. The accuracy of a SaMD cannot be defined by a single measure, but is instead a combination of factors assessed during its development and validation. The primary factor is how well the SaMD fulfils its intended purpose, such as the accuracy with which a SaMD designed to detect polyps during colonoscopies identifies such lesions [54]. Clinical validation requires the SaMD to be tested with real-world data from clinical studies, comparing its outputs to established diagnostic methods [55]. Specific metrics can be used to assess accuracy, depending on the SaMD’s function: for image analysis tools, this includes the ability to correctly identify true positives (sensitivity) and to correctly identify true negatives (specificity) [56]. In addition, an accurate SaMD should produce consistent results when applied to similar datasets, regardless of the user or setting. The requirements for accuracy might be established based on the SaMD’s risk category, with higher-risk SaMDs requiring more stringent validation and higher thresholds of accuracy. Overall, the accuracy of SaMDs is multi-faceted, ensuring the software performs its intended medical function reliably and that it delivers results aligned with established diagnostic methods.

When considering the reliability of SaMDs, it is necessary to contemplate different issues to ensure SaMDs perform as expected and minimize risks to patients. SaMDs must perform consistently, repeatedly producing the same result for a given input, regardless of the user or environment [57]. In addition, a reliable SaMD should have a low failure rate, rarely malfunctioning or producing incorrect outputs, and it must be available for use when needed, with minimal downtime for maintenance or due to technical issues [33,58]. Along similar lines, reliability also involves recovery mechanisms, with minimal data loss or disruption to patient care in the case of failure, possibly through automatic back-ups or fail-safe modes to ensure data integrity even in the case of errors [57]. Indeed, regulatory bodies establish accuracy and reliability requirements based on the SaMD’s risk classification, with stricter demands placed on higher-risk devices (those involved in critical diagnoses or treatment decisions) [56,59]. These features must be anticipated in the design and testing phases [33,56], and SaMDs must incorporate robust cybersecurity to protect from cyberattacks that could compromise functionality or patient data integrity [35,60]. In addition, the common lack of interoperability in clinical systems necessitates the development of mapping between various standards, or even proprietary systems, to facilitate data access.

### 6.2. Clinical Barriers Including Clinician Adoption, Training Needs, and Patient Acceptance

Beyond development/technical issues, several other challenges may hinder SaMD uptake into clinical workflows. One challenge is related to their ability to communicate and integrate with different systems used to store and access clinical data (e.g., EHRs), and even their capacity to communicate and integrate with other SaMDs. A failure in this sense can make it challenging to gain a full vision of patient health, necessary to achieve the maximum benefit from these tools [33,58]. The development of harmonized and interoperable frameworks and protocol is mandatory to ensure the effective integration of SaMDs and HER in clinical practice.

Beyond interpretability [41,42,61,62], explainability [63,64] is also important when implementing SaMDs. An explainable device can be readily understood [65], avoiding “black-box medicine” when it is unclear how clinical decisions are made [66]. The responsibility of clinicians for their decisions implies they can usually provide coherent explanations as required. Some lack of explainability can be tolerated in medicine—aspirin, for example, was prescribed as an analgesic for almost a hundred years without the full understanding of its mechanism [67]. This raises the question: can SaMDs be used without fully understanding how they reach a conclusion? As patients become more involved in their own healthcare, the demands on clinicians to be able to explain recommendations increases, especially as shared decision-making becomes more frequent. Moreover, some level of understanding is needed for physicians to recognize the limits of these tools and when they might be acting irregularly. Explainable AI models can be important to the physician’s understanding of which region is most likely to contain a lesion or be classified into a specific category [45,68].

The introduction of SaMDs and AI tools into medicine also impacts the attribution of responsibility for treatment and adverse outcomes [45]. There is currently a gap in the determination of legal responsibility if SaMD use causes harm [69], making it difficult to establish autonomy and agency [70], and whether errors or failures have occurred. Responsibility when using SaMDs is typically shared between physicians and their institutions, but what about the developers? While the physician may be held accountable, perhaps no one acted improperly, or the SaMD behaved unexpectedly. In cases where SaMD performs its tasks reliably, but still fails, the clinician may be forced to take responsibility for decisions they did not directly make. This uncertain responsibility may affect the patient’s trust in their clinician [71], particularly as clinicians increasingly rely on SaMDs, shifting trust towards the tool itself [45]. Additionally, implementing these tools may raise concerns about the clinician’s role and fears that they will be “replaced” by technology [72], although, ideally, clinicians should leverage these advances to enhance their practice.

SaMDs have the potential to optimize personnel organization [73], and to free-up clinicians [50] or enhance their capacity to attend to patients. However, the question arises: should we look to use fully automated SaMDs? Could negative findings from GI examinations with these tools be directly communicated to the patient? These issues challenge the patient–clinician relationship and may contribute to the dehumanization of healthcare, which could be detrimental given the therapeutic value of human attention and empathy [74,75]. While automated systems may enable clinicians to spend more time with patients, their ability to interpret endoscopy images or identify more complex changes or subtle changes may be compromised (also known as “de-skilling”) [65]. On the other hand, clinicians might review more images with the aid of automated workflows, thus rapidly fine-tuning their skills through exposure to a high proportion of clinically relevant images, but perhaps with reduced exposure to normal images. A downside of increased automation is that clinicians might become more inclined to accept the decisions of these tools, even when they may be incorrect [50,76,77], potentially influencing diagnostic precision. Thus, the incorporation of SaMDs must be accompanied by efforts to ensure clinicians remain well trained and that they do not become dependent on automated systems [42,63,76,78].

Other concerns include the influence that non-medical professionals (e.g., computer scientists and IT specialists) may exert in healthcare settings, and the possible appearance of monopolies in specific healthcare areas due to a reliance on software [45].

## 7. Future Trends and Innovations

### 7.1. Predictive Analytics and Their Potential to Revolutionize Preventive Care in Digestive Health

Predictive analytics involve applying statistical techniques in conjunction with ML algorithms to extract patterns from data that can then be used to predict future events. These patterns may be difficult to perceive by human observation and may not even be discerned through epidemiological studies. This is a powerful tool in preventive healthcare, and in digestive medicine it can help the early detection of specific conditions and possible flare-ups of others like IBD, enabling adjustments to medication or lifestyle. This approach can enhance personalized care by considering the individual’s clinical history, lifestyle, and factors like age, weight, diet, family history, and gut microbiome to evaluate their health risks. Early signs of many digestive conditions are often difficult to detect, so conducting predictive analyses on the basis of data from devices like health trackers or smart toilets is crucial for detecting subtle changes and raising the alarm for early detection and treatment.

Moreover, predictive models can help design personalized diets, exercise routines, or even supplements to promote gut health, reducing the risk of future health problems. These approaches face similar challenges regarding data privacy and security, and the accuracy of these models again depends on the quality and quantity of the data available. In this context, the number of potentially underrepresented cases in the data could be increased by integrating blockchain-based solutions into healthcare platforms, enhancing the training and successful implementation of SaMDs.

### 7.2. The Role of Cross-Disciplinary Collaborations for Nurturing Innovation

The nature of SaMDs means cross-disciplinary collaborations, which are fundamental for driving innovation in these tools. SaMDs bridge the fields of medicine and software engineering, intersecting with other areas such as data science, user experience design, technology assessment, and regulation, each with its own knowledge base and terminology. Effective collaboration requires clear communication regarding the needs and clinical contexts to be translated into technical solutions. The odds of success in these collaborations are greatly increased when teams have a wide mindset, paving the way to greater innovation. Again, safety and regulation demands will bring in specialists from other areas, further enhancing the cross-fertilization of ideas.

Thus, teams involved in developing SaMDs for digestive healthcare are likely to include: clinicians specializing in digestive medicine, healthcare management experts ensuring seamless device integration into clinical workflows, software engineers designing robust and secure SaMDs following best practices, and regulatory experts guiding the development process and patients to ensure the tools effectively meet their needs and preferences. The communication and collaboration between these diverse groups will lead to the development of safer, more effective, and user-friendly tools that will improve patient care. Additionally, the widespread adoption of SaMDs in digestive healthcare presents an opportunity for the secondary use of collected images, potentially generating new evidence from the original data.

## 8. Conclusions

The transformative potential of Software as a Medical Devices (SaMDs) to reshape digestive healthcare is evident, particularly in the realm of personalized medicine. These devices may significantly improve diagnosis, treatment, and patient management by incorporating AI algorithms, which allows the leveraging of a vast amount of data currently available, ultimately enhancing accuracy and performance.

The development and implementation of SaMDs highlights the need for adequate regulatory frameworks to ensure that these tools remain transparent in their functioning and comply with the FAIR principles for data management: findability, accessibility, interoperability, and reusability. SaMDs must also be adapted for use with different devices and in different settings, incorporating data from diverse centres. This adaptability, however, increases the demands for compliance with data protection guidelines and the establishment of adequate security measures, which are critical for its successful integration into clinical workflows.

This study also emphasizes that future advances should address the need for adequate clinical studies and real-world data to demonstrate the safety and suitability of these devices to healthcare settings. Additionally, as the field evolves, progress may shift towards a transition from semi-automated to a more fully automated analysis, further enhancing the capabilities of SaMDs in digestive healthcare.

## Figures and Tables

**Table 1:** Examples of emerging technologies currently being developed as SaMDs in digestive Healthcare.

Areas of GI Action	Challenges	AI-Based SaMD	Potential Applications
Capsule Endoscopy	The extensive duration of capsule endoscopy videos (specifically if considering capsule panendoscopy) leads to high reading burden, increasing the probability of missed diagnoses	Automated video analysis software to highlight areas of interest, reducing manual review time	Detection of gastrointestinal bleeding, monitoring inflammatory bowel disease, and colorectal cancer (and panendoscopy) screening
Cholangioscopy	Differentiating benign from malignant biliary strictures is complex, with a significant inter-observer variability and low biopsy precision, even with image guidance	Providing real-time malignancy probability and lesion localization heatmaps	Improved characterization of biliary strictures, potentially aiding in the diagnosis of high-risk patients (e.g., primary sclerosing cholangitis)
Endoscopic Ultrasound	Identification and characterization of pancreatic lesions are challenging, with limited accuracy	Real-time diagnostic suggestions and malignancy risk assessment	Enhanced evaluation of pancreatic lesions, potentially improving treatment planning
Device Assisted Enteroscopy	Unique diagnostic and therapeutic properties for comprehensive GI tract assessment	Identification and classification of lesions during the procedure	Facilitates differential diagnosis and guides therapeutic interventions
High Resolution Anoscopy	Assessing benign and precancerous anal canal lesions is difficult, requiring significant expertise and long learning curve	Real-time detection and differentiation of high-grade squamous intraepithelial lesions	Enhances evaluation of anal canal lesions, making the treatment of precancerous lesions more efficient
High Resolution Manometry	Achieving consistent and accurate digestive motility assessments is challenging due to technical complexity and varied interpretation skills	Streamlines diagnostic protocol execution, and interprets motility patterns with accuracy	Improves diagnosis and management of digestive motility problems

**Table 2:** Distinction between SaMD categorization classes is based on their purpose, whether for information management, driving decisions, or treatment/diagnosis.

	Class I	Class II	Class III	Class IV
Informs	NA Only retrieves information, and organises and optimizes data	Serious Provides important information, although it does not lead to major decisions	Critical Provides important information, including urgent situations	Critical Provides important information, including urgent or life-threating situations
Drives	NA	Non-Serious Aids decisions for minor health concerns, without significant impact	Serious Aids decisions for significant, non-life-threatening concerns	Critical Aids decisions for significant, life-threatening concerns
Treat/Diagnoses	NA	Non-Serious Assists with minor health concerns	Serious Assists with significant, non-life-threatening concerns	Critical Assists with urgent, life-threatening conditions
Regulatory Requirements	Mimimal regulatory oversight	Some pre-market review	Significant pre-market review	Extensive pre-market

## Data Availability

No new data were created or analyzed in this study.
